# Regulation of Alpha-Secretase ADAM10 *In vitro* and *In vivo*: Genetic, Epigenetic, and Protein-Based Mechanisms

**DOI:** 10.3389/fnmol.2017.00056

**Published:** 2017-03-17

**Authors:** Kristina Endres, Thomas Deller

**Affiliations:** ^1^Clinic of Psychiatry and Psychotherapy, University Medical Center Johannes Gutenberg-University MainzMainz, Germany; ^2^Institute of Clinical Neuroanatomy, Neuroscience Center, Goethe-UniversityFrankfurt/Main, Germany

**Keywords:** ADAM10, aging, alpha-secretase, Alzheimer's disease, mouse models, promoter, transcription factors, spine

## Abstract

ADAM10 (A Disintegrin and Metalloproteinase 10) has been identified as the major physiological alpha-secretase in neurons, responsible for cleaving APP in a non-amyloidogenic manner. This cleavage results in the production of a neuroprotective APP-derived fragment, APPs-alpha, and an attenuated production of neurotoxic A-beta peptides. An increase in ADAM10 activity shifts the balance of APP processing toward APPs-alpha and protects the brain from amyloid deposition and disease. Thus, increasing ADAM10 activity has been proposed an attractive target for the treatment of neurodegenerative diseases and it appears to be timely to investigate the physiological mechanisms regulating ADAM10 expression. Therefore, in this article, we will (1) review reports on the physiological regulation of ADAM10 at the transcriptional level, by epigenetic factors, miRNAs and/or protein interactions, (2) describe conditions, which change ADAM10 expression *in vitro* and *in vivo*, (3) report how neuronal ADAM10 expression may be regulated in humans, and (4) discuss how this knowledge on the physiological and pathophysiological regulation of ADAM10 may help to preserve or restore brain function.

## ADAM10 - portrait of a biologically versatile protease

### Introduction

ADAM10 (A Disintegrin and Metalloproteinase 10) was identified *in vitro* as a key proteinase in the processing of the amyloid precursor protein (APP) more than 15 years ago (Lammich et al., 1999). The zinc-dependent proteinase cleaves APP within the A-beta sequence, thus preventing the production of this peptide. Furthermore, APP-cleavage by ADAM10 liberates APPs-alpha, which has neuroprotective properties and is involved in the regulation of synaptic plasticity and learning and memory (reviewed in Kögel et al., 2012). In line with these findings, overexpression of ADAM10 in mice revealed elevated APPs-alpha levels and demonstrated a robust *in vivo* activity of ADAM10 (Postina et al., 2004). Overexpression of ADAM10 was also effective in animal mouse models of Alzheimer's disease (AD) and reduced plaque load as well as deficits in learning and memory (Postina et al., 2004; Schmitt et al., 2006). Subsequent investigations of RNAi-mediated knock-down of the enzyme in primary cortical neurons (Kuhn et al., 2010) as well as conditional knock-down in mice (Jorissen et al., 2010) consolidated the enzymes' role in APP processing *in vivo*. Collectively, these data point to ADAM10 as being the most important physiological alpha-secretase involved in the processing of APP in neurons.

The central role of ADAM10 in APP processing has made ADAM10 an interesting target for AD therapy. It has been proposed (e.g., Fahrenholz and Postina, 2006; Vincent and Govitrapong, 2011) that similar to the situation in intact animals (Postina et al., 2004) an increase in ADAM10 could result in decreased A-beta load and improved learning and memory in AD patients. For this approach to be effective and safe, however, the cell biology of ADAM10 and its cellular functions need to be better understood. ADAM10 is a versatile protease which cleaves not only APP but also several other proteins (see paragraph 2). Therapeutic strategies for AD focusing on ADAM10 as a target have to keep these additional substrates in mind. In the present review we will summarize the extant literature on ADAM10 and focus on what is known about its regulation *in vitro* and *in vivo*. Understanding the regulation of this enzyme may be a necessary step toward understanding its usefulness in therapeutic contexts.

### Domain structure, cellular synthesis, and maturation of ADAM10

**Figure 1:**
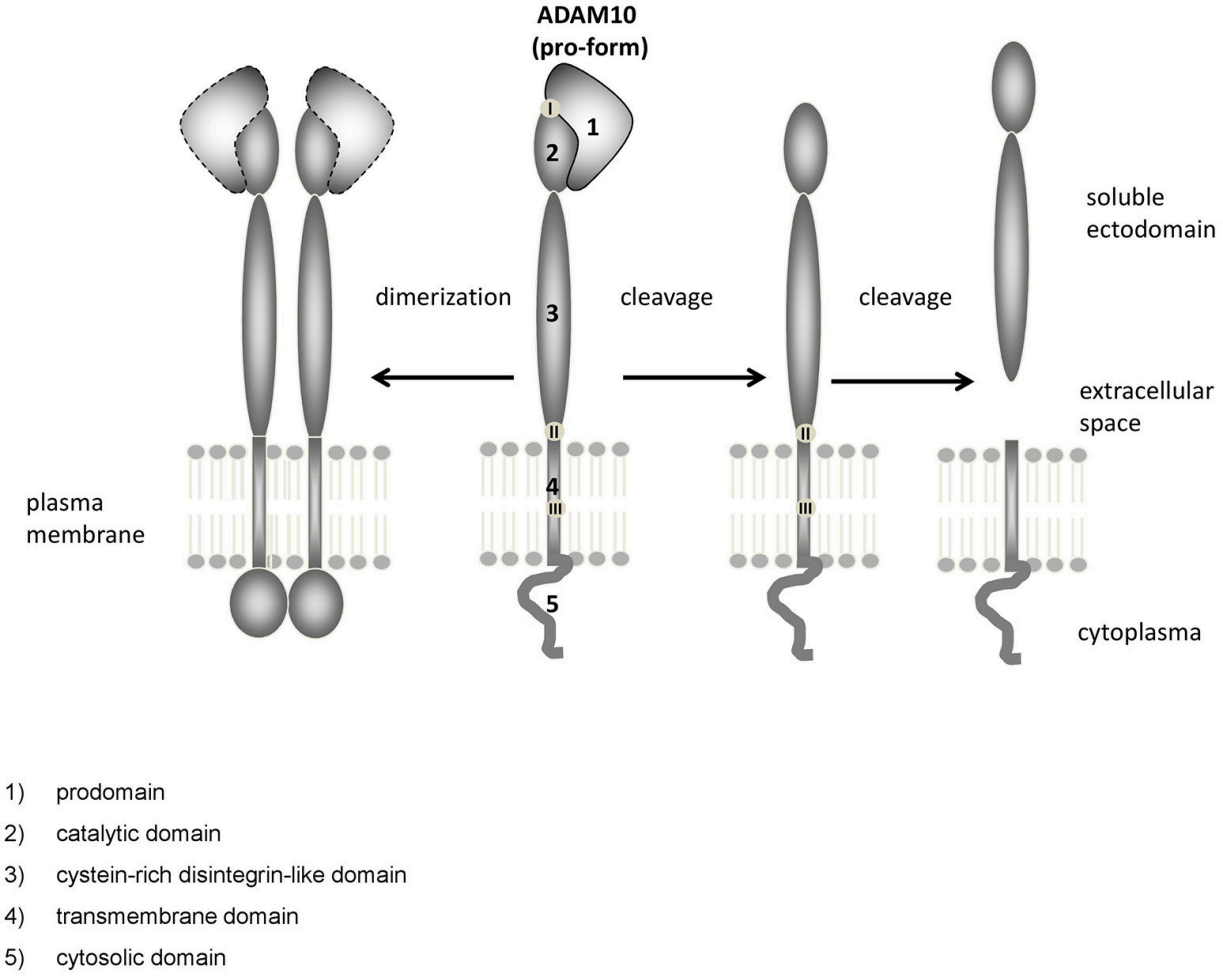
**Domain structure of ADAM10**. ADAM10 consists of several functional distinct domains: (1) prodomain, (2) catalytic domain, (3) cystein-rich disintegrin-like domain, (4) transmembrane domain, (5) cytosolic domain. Upon dimerization (left), the unstructured C-terminus converts into an ordered domain (Deng et al., 2014). Cleavage sites for proteinases such as proprotein convertases [located at the end of the prodomain, (Anders et al., 2001), I], other ADAMs [close to the membrane, (Cissé et al., 2005; Parkin and Harris, 2009; Tousseyn et al., 2009) II] or gamma-secretase [within the membrane, (Tousseyn et al., 2009), III] have been identified.

ADAM10 is a catalytically active member of the ADAM family of proteinases. The ADAMs are grouped together as a family because they share structural features with snake venom disintegrin proteases (Wolfsberg et al., 1995a,b). ADAM10 is co-translationally synthesized via the rough ER, matures and is transported via the Golgi apparatus. Maturation includes removal of the prodomain (Figure 1: 1), which keeps the enzyme in an inactive state. A cleavage site for proprotein convertases such as PC7 (Anders et al., 2001) is mandatory for production of the catalytically active enzyme as shown by analyzing mutated ADAM10. However, the prodomain has not a mere inhibitory function but is also needed as an intramolecular chaperon for correct folding (Anders et al., 2001). This is reflected by the fact that a large proportion of ADAM10 has been found to be localized in the Golgi apparatus in AR breast carcinoma cell line by confocal microscopy (Gutwein et al., 2003). The mature form of ADAM10 of about 68 kDa was found in the Golgi compartment as well as in the ER/plasma membrane-enriched fraction of postnuclear supernatant and at least cleavage of another substrate of ADAM10—the L1 adhesion molecule—seems to occur in both. Recent investigations suggested by administering the inhibitor RVKR for up to 8 h before measuring shedding activity that cleavage by proprotein convertases might be dispensable for rapid stimulation of ADAM10 (Maretzky et al., 2015). However, as the half-life time of ADAM10 is rather long (>72 h; Mezyk-Kopec et al., 2009), this result may need to be interpreted with some caution.

The catalytic domain of ADAM10 (Figure 1: 2) contains the characteristic zinc-binding consensus motif (HEXGHXXGXXHD) of active members of the proteinase family. A point mutation within this motif (E384A) results in a dominant negative acting protein and a decreased APPs-alpha secretion, as could be shown shown in HEK cells and mice (Fahrenholz et al., 2000; Postina et al., 2004).

The catalytic and the proximal disintegrin domain contain high-mannose as well as complex-type N-glycan attachment sites (Escrevente et al., 2008). The disintegrin domain of ADAM10 (Figure 1: 3) does not appear to be essential for ADAM10 protease activity in cell culture experiments (Fahrenholz et al., 2000). Rather, the short intracellular C-terminus seems to play an important role: Epidermal growth factor (EGF) cleavage has been reported to be partially impaired in ADAM10−/− cells overexpressing a cytoplasmic domain deletion mutant of the proteinase (Horiuchi et al., 2007). However, the cytoplasmic domain of ADAM10 has also been reported to negatively influence constitutive shedding through an ER retention motif: an ADAM10Δcyto mutant displayed increased catalytic activity compared to ADAM10 Wt with regard to Betacellulin cleavage (Maretzky et al., 2015). The cytoplasmic domain of ADAM10 contains several binding sites that may be involved in regulatory events, such as an IQ consensus binding site for calmodulin (Horiuchi et al., 2007) and two proline-rich putative Src homology 3 (SH3) binding domains. The juxtamembrane binding site affects basolateral localization of ADAM10 in epithelial cells (Wild-Bode et al., 2006), while in neurons the SH3 binding domains direct ADAM10 to the postsynaptic membrane (Marcello et al., 2007). Using a phage library analysis comprising 305 human SH3 domains, 38 candidate binding proteins for the ADAM10 C-terminus were identified, including endophilin-A2, Lck, or ZDHHC6 (Ebsen et al., 2014). Although the biological relevance of many of these putative ADAM10 binding partners needs to be determined, this finding suggests that regulatory interactions at the C-terminus could play a major role regarding the cellular localization as well as the activity of the proteinase.

### Developmental and adult expression of ADAM10 in mouse and human brain

ADAM10 is expressed in various tissues in mice (Marcinkiewicz and Seidah, 2000). Its presence in the developing as well as in the adult CNS underscores its importance for normal brain development and function. Since ADAM10 can only process a putative substrate if both, protease and substrate are expressed at the same time and in the same cellular compartments, it is important to know the temporospatial pattern of ADAM10 expression in the brain. This pattern can then be compared to corresponding data of putative substrates or binding partners.

**Figure 2:**
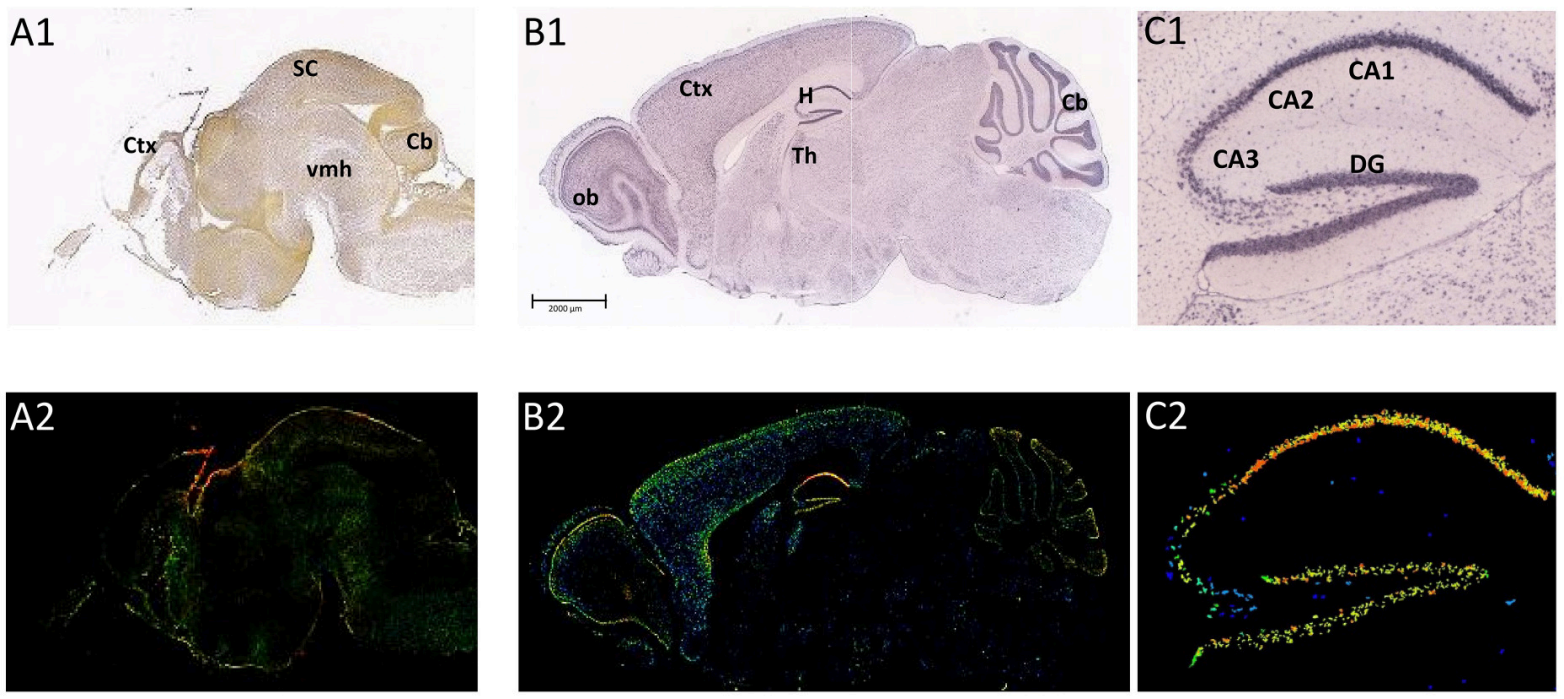
**Distribution of ADAM10 mRNA in the murine brain**. Sagittal section of C57Bl6/J mouse brain (male) at E18.5 (**A1,A2**; Image credit: Allen Institute; http://developingmouse.brain-map.org/experiment/show/100055949, ©2016. Allen Institute for Brain Science) and P56 (**B1,B2**; Image credit: Allen Institute; http://developingmouse.brain-map.org/experiment/show/69514738, ©2016. Allen Institute for Brain Science **C1,C2**: magnification of hippocampal area of the adult brain). ADAM10 mRNA expression is revealed by *in situ* hybridization [**A1–C1**, upper row ISH; **A2–C2**, lower row expression energy (cells with highest probability of gene expression)]. CA1-3, *Cornu Ammonis* regions; Cb, cerebellum; Ctx, cerebral cortex; DG, *Dentate Gyrus*; H, hippocampus; ob, olfactory bulb; SC, Superior Colliculus; Th, thalamus; vmh, ventral mid-/hindbrain

The distribution of ADAM10 was studied in mouse cerebral cortex from embryonic day (E) 14.5 to postnatal day (P) 1 using *in situ* hybridization analysis. This revealed ADAM10 expression within the ventricular zone and the cortical plate from E17.5 to P1 (Ma et al., 2013; see also Figure 2). These data on ADAM10 mRNA were corroborated by immunofluorescence analyses which detected ADAM10 protein in developing cerebral cortex from E14.5 to E18.5 (Ma et al., 2013).

ADAM10 plays an essential role during development. Animals with a conventional ADAM10 knock-out die on E9.5 (Hartmann et al., 2002), which underlines the general importance of this protease. More recently generated conditional Nestin-Cre-ADAM10 knock-out mice with a cell-specific inactivation of ADAM10 in neural progenitor cells (NPCs), NPC-derived neurons and glial cells prolonged the life span of the mice to a perinatal time point (Jorissen et al., 2010). These mutants displayed a disrupted neocortex and a severe reduction of the ganglionic eminence. Knock-out of ADAM10 in the postnatal CNS using a CaMKII-alpha-Cre driver finally allowed investigation of adult mice (Prox et al., 2013). This conditional mutant did not show gross morphological abnormalities but exhibited synaptic dysfunction, increased early perinatal lethality, altered behavior, and epileptic seizures. Similar results were reported by another group which independently established an adult ADAM10 knock-out model (Zhuang et al., 2015). Taken together, these studies indicate that ADAM10-deficiency results in major developmental phenotypes. Lack of the protease at later stages is compatible with life but results in a number of dysfunctions.

The cellular expression pattern of ADAM10 was also investigated in some of these studies. Interestingly, ADAM10 protein expression partially correlated with both, S100β and Tuj1 expression (E16.5 to P1), which indicates a relevance of ADAM10 for glial as well as neuronal cell function during late embryonic cerebral cortex development stages (Ma et al., 2013). In the developing brain of chicken, ADAM10 shows a weak but widespread expression at E12 in most gray matter areas (Lin et al., 2008). Expression intensity decreased from E14 to E19, with the exception of the telencephalon and the cerebellum. Some ADAM10-positive non-neuronal cells may be oligodendrocytes, since they were shown to co-express galactocerebroside, which is a marker for oligodendrocytes at late stages of chicken embryogenesis (Lin et al., 2008).

ADAM10 expression has also been studied in developing human brain: Bernstein and colleagues compared the amount of ADAM10 in temporal cortex of stillborn children with those of normal aged adults and found a general increase (Bernstein et al., 2003). In a follow-up study, a weak expression of ADAM10 in the cytoplasm of pyramidal as well as non-pyramidal neurons was confirmed in pre- and perinatal human brains (Bernstein et al., 2009). In contrast to these findings, analysis of total human fetal brain RNA obtained from a commercial source (Clontech, Kaczur et al., 2007) failed to detect a prominent ADAM10 expression using microarray analysis. Differences in the stage of fetal development (which has not been reported) or technical issues, e.g., detection of ADAM10 in total mRNA preparations, may explain these discrepancies. Analysis of transcripts from a human fetal brain library, however, revealed two types of ADAM10 cDNAs: one encoding a 748 amino acid protein [designated Kuzbanian (Kuz)L] and a second one (KuzS, encoding a 568 amino acid protein), which lacks the cysteine-rich, transmembrane, and cytoplasmic domain (Yavari et al., 1998). Fetal human brain expressed substantially more of the short than of the long variant, while fetal lung predominantly contained the longer variant. In adult human brain tissue, Northern blot demonstrated the persistence of both forms in different amounts (Yavari et al., 1998). The presence of the short transcript appears to be unique to humans as the transcript could not be detected in adult and embryonic tissue of mice. Whether it is functionally relevant, i.e., whether it is translated into a biologically active protein is unclear.

The expression of ADAM10 in the adult brain has been studied in rodents and humans. By using northern blot technique it could be shown that adult human amygdala, caudate nucleus and corpus callosum contain relatively high amounts of ADAM10 transcripts whereas mRNA levels in the subthalamic nucleus and the thalamus were comparably low (Yavari et al., 1998). In the adult rodent brain, ADAM10 mRNA was reported to be moderately expressed throughout the whole brain, including the olfactory bulb, the hippocampus or the subthalamic region (mouse and rat: Kärkkäinen et al., 2000, see also Figure 2: P56). Semiquantitative evaluation of ADAM10 mRNA using ISH analysis revealed only the Pontine nuclei as a brain structure not expressing the protease (Kärkkäinen et al., 2000). These findings have been confirmed by a recent investigation that also described positive ISH-stainings for neurons of the cerebral cortex, hippocampus, thalamus, and cerebellar granular cells in the CNS of adult mice (Guo et al., 2016).

## ADAM10—physiological substrates and functions

### Physiological substrates of ADAM10

ADAM10 is probably best known for its ability to process APP. ADAM10 cleaves APP at the alpha-secretase cleavage site and *in vitro* as well as *in vivo* studies have implicated ADAM10 as the biologically most relevant neuronal alpha-secretase (e.g., Postina et al., 2004; Jorissen et al., 2010; Kuhn et al., 2010). Of note, the regional and cellular overlap of ADAM10 and APP, which is necessary for ADAM10 to process APP in tissues, is age-dependent: at early developmental stages the mRNA distributions of ADAM10 and APP are not fully congruent but with aging the overlap increases (Marcinkiewicz and Seidah, 2000). This finding—but also the wealth of data on other substrates of ADAM10 (see below)—suggests that ADAM10 substrates may change: During development and in the young brain ADAM10 may preferentially cleave substrates other than APP and the role of ADAM10 as alpha-secretase of APP may emerge with aging.

Presently, a rather large number of ADAM10 substrates have been identified in different experimental settings (e.g., reviewed for proteomic approaches in Müller et al., 2016). Of notice, ADAM10 substrates belong to type I as well as type II transmembrane but also Glycosylphosphatidylinisotol (GPI)-anchored proteins, indicating a considerable flexibility of the protease with regard to substrate recognition. Consensus cleavage motifs for proteases are commonly deduced from the amino acids surrounding the naturally occurring cleavage sites within protein substrates. This approach failed in the case of ADAM10 because it lacks a well-defined consensus sequence: for ADAM10 leucine was found to be preferred (and tyrosine accepted) in the P1' position (immediately downstream of the cleavage site) in an investigation using oriented peptide mixture libraries which gives evidence of a shallow or deep S1' site (John et al., 2004). ADAM10's preference for larger residues at P1' has been confirmed but acceptance of aromatic amino acids and even glutamine were also reported (Caescu et al., 2009). This tolerance for aromatic residues in P1' may be the most relevant difference in cleavage site specificities between ADAM10 and its close relative ADAM17 (Tucher et al., 2014). In the early investigation tyrosine was found to be favored at P1 (immediately upstream of the cleavage site; John et al., 2004). In contrast to this, selectivity for small residues such as alanine at the P1 was described by Caescu et al. (2009) and specificities for proline and basic residues were recently reported (Tucher et al., 2014). In sum, these reports show the methodological limitations and uncertainties involved in pinpointing cleavage site specificities from linear unmodified peptide libraries. In addition, the activity state of the cell may also influence shedding capacity, as is the case for NG2 (Sakry et al., 2014) as well as the synaptic marker neuroligin 1 (Suzuki et al., 2012), further complicating work in this direction.

At present, a wide variety of substrates has been identified for ADAM10 and some of them have been confirmed not only in primary culture but also *in vivo*. In line with its ubiquitous expression, ADAM10 substrates are linked to a number of biological systems and physiological as well as pathological functions (c.f. chapter 4), including the immune and nervous system but also cancerogenesis (e.g., Vincent and Checler, 2012). In their review Pruessmeyer and Ludwig reported on the “good, the bad and the ugly” ADAM10 substrates (Pruessmeyer and Ludwig, 2009). Since then, a number of additional ADAM10 substrates were identified and have resulted in a more complete, albeit even more complex picture of ADAM10 (Table 1).

**Table 1:** **ADAM10 substrates identified within the last years**.

**Protein**	**Type**	**Evidence**	**References**	**Expression**
Leda-1/Pianp	Type I membrane protein	Cell culture, MEFs (no distinction between ADAM10 and 17)	Biswas et al., 2016	CNS cells, murine melanoma cell line B16F10 and rat liver sinusoidal endothelial cells
gp130	Type I membrane protein	Cell culture	Wolf et al., 2016	Ubiquitous
IL-11R	Type I membrane protein	Cell culture, MEFs	Lokau et al., 2016	e.g., bone, heart, lung, spleen, gastrointestinal tract, and uterus
LDLR MT4MMP LRRC4B NRCAM NEO1 CNTN2 (only substrates validated by immunoblot are included)	Type I membrane protein GPI anchored (CNTN2)	ADAM10 ko neurons	Kuhn et al., 2016	Diverse
NKG2D MIC ligands	Type I membrane protein	Cell culture, plasma cells	Wolpert et al., 2014; Zingoni et al., 2015	Induced by different types of stress in cells
SIRPα	Type I membrane protein	Cell culture	Londino et al., 2015	Monocyte lineage and neuronal cells
TACI	Type III membrane protein	Cell culture, B-cells	Hoffmann et al., 2015	Activated B-cells
NG2	Type I membrane protein	Cell culture, acute brain slices, isolated OPC	Sakry et al., 2014	Glia lineage
FAT1	Type I membrane protein	Cell culture	Wojtalewicz et al., 2014	Various tissues, upregulation in cancer
TREM2	Type I membrane protein	Cell culture	Kleinberger et al., 2014	Microglia
Cad6B	Type II membrane protein	Cell culture, neural crest cells	Schiffmacher et al., 2014	E.g. neural crest cells
CD154	Type II membrane protein	Cell culture	Yacoub et al., 2013	T cells
Coxsackievirus and Adenovirus Receptor (CAR)	Type I membrane protein	Cell culture	Houri et al., 2013	Highly expressed in the developing nervous system
Neuroligin 1	Type I membrane protein	Primary neurons	Suzuki et al., 2012	Synaptic
Cell adhesion molecule 1 (CADM1)	Type I membrane protein	Cell culture	Nagara et al., 2012	Various tissues
Annexin A1		Cell culture	Blume et al., 2012	Necrotic cells
Alcadeins	Type I membrane protein	Cell culture, MEFs	Hata et al., 2009	Neuronal
collagen XVII/BP180	Type II membrane protein	Primary keratinocytes, MEFs	Franzke et al., 2009	Skin
Pmel17	Type I membrane protein	Cell culture	Kummer et al., 2009	Melanocytes and retinal epithelial cells
Klotho	Type I membrane protein	Cell culture, MEFs	Bloch et al., 2009	Predominantely in kidney and in brain in the choroid plexus
C4.4A	GPI anchored	Cell culture	Esselens et al., 2008	Various tissues, upregulation in cancer
Bri2 (ITM2b)	Type II membrane protein	Cell culture	Martin et al., 2008	Brain

*Putative ADAM10 substrates (ordered from newest to oldest publication date) identified since 2009 or not included in Pruessmeyer and Ludwig (2009) are listed. (PubMed search 29-11-2016: “ADAM10 and substrate” or “ADAM10 and proteolysis”)*.

### Functions of ADAM10 at the synapse and in non-neuronal cells

ADAM10 processes other proteins and thus, influences the functions of its substrates by in-/activating them or by liberating biologically active fragments. Thereby, the biological effects of ADAM10 activity are tightly linked to the functions of the substrates and their cleavage products. Because of the large number of ADAM10 substrates identified to date, we focus in this review on those which are known to have an important impact on brain function and which are likely to co-localize with ADAM10 at the synapse or in glial cells.

The earliest study on the distribution of ADAM10 at synapses was based on immunocytochemistry and suggested that ADAM10 co-localizes with the postsynaptic scaffold protein Synapse-associated protein 97 (SAP-97) but not with the presynaptic vesicle protein synaptophysin (Marcello et al., 2007). However, a more recent study using the sensitive proximity ligation assay reported proximity of the enzyme with synaptophysin in mouse primary hippocampal neurons (Lundgren et al., 2015). This suggests that ADAM10 can be present in both parts of a synapse. One example where this could be functionally relevant is the neurexin-neuroligin-interaction: neurexins and neuroligins are cell-adhesion molecules which form transsynaptic complexes (e.g., Tsetsenis et al., 2014). They appear to be important for normal synapse specification and function (Jedlicka et al., 2011, 2015). For the postsynaptic protein Neuroligin 1, ADAM10 has been found to act as the major sheddase, as could be shown by pharmacological and genetic means in primary rat cortical neurons (Suzuki et al., 2012). NMDA receptor activation as well as prolonged epileptic seizure condition increased shedding, suggesting a role for neuronal activity in this context. Interestingly, shedding of Neuroligin 1 could be induced by soluble neurexin 1α or β derived from overexpressing HEK293 cells (Suzuki et al., 2012), indicating that ligand binding at the cell surface also regulates Neuroligin 1 shedding. Similar observations have been made for the Notch-Delta complex where Notch1 cleavage by ADAM10 is induced by Delta binding (e.g., reviewed in Van Tetering and Vooijs, 2011). Intriguingly, Notch 1 as well as its ligands - Delta or Jagged - have been found to be cleaved by ADAM10 (for example: Pan and Rubin, 1997; Lavoie and Selkoe, 2003). A recent publication regarding systemic characterization of ADAM10 substrates from neurons highlighted that ADAM10 is also in principle capable of shedding the Neuroligin ligands Neurexins 2 and 3, although deletion of the proteinase resulted only in a comparably mild reduction of the shedding (Kuhn et al., 2016). If this role for ADAM10 in the cleavage of major anchoring proteins can be verified *in vivo* and in human brain, interfering with ADAM10 activity may indeed be a powerful tool to influence synaptic structure and function.

ADAM10 has also been found to process substrates of non-neuronal cells. Since neurons and glial cells are highly interdependent and jointly regulate synapse functions, ADAM10 may also influence network activities through glial cells. For example, the marker transmembrane proteoglycan nerve-glia antigen 2 (NG2), commonly found on the so-called “NG2-glial cells” (Eugenín-Von Bernhardi and Dimou, 2016), has also been identified as a substrate of ADAM10 (Sakry et al., 2014). Similar to what has been reported for Neuroligin 1, shedding of NG2 is also regulated by neuronal activity. Moreover, neurons from NG2-knock-out mice exhibited diminished amplitudes of AMPA receptor-currents which could be rescued by application of the partial NG2 ectodomain (Sakry et al., 2014). This suggests that an NG2-cell derived ectodomain produced by ADAM10 processing regulates synaptic activity and plays a role in neuron-glia communication.

Another non-neuronal substrate of ADAM10 with implications for glial and neuronal function is the microglial surface protein triggering receptor expressed on myeloid cells 2 (TREM2). TREM2 has been suggested to play a role in phagocytosis and has been recently recognized as a genetic risk-factor for AD (Frank et al., 2008; Colonna and Wang, 2016). Using HEK293 Flp-In cells and enzymatic inhibitors, the release of soluble TREM2 ectodomain was demonstrated to depend on ADAM10 but not on ADAM17 or beta-secretase (beta-site amyloid precursor protein cleaving enzyme 1; BACE1) (Kleinberger et al., 2014). Reduction of cell surface TREM2 decreases the ability of microglia to phagocytose and remove cellular debris or apoptotic neurons (Kleinberger et al., 2014). Of note, TREM2 ligands were identified on Neuro2A cells and on cultured cortical and dopamine neurons (Hsieh et al., 2009), suggesting an impact of a non-neuronal shedding event on neurons.

Finally, there is growing evidence for a role of exosomes in neuron-glia communication (Frühbeis et al., 2013). In this regard it is of interest that functionally active ADAM10 has been found in exosomes from ovarian carcinoma cells where it contributes to L1 and CD44 cleavage (Stoeck et al., 2006) and in exosomes of primed B-cells (Padro et al., 2013). Whether microglial or neuronal cells also use exosomes to deliver ADAM10 or shedded substrates among themselves is currently unknown.

## Regulation of ADAM10

ADAM10 is a multifunctional protease active throughout the life of an organism and its regulation is controlled at transcriptional, epigenetic, translational and post-translational levels. These different levels of regulation allow a cell to adapt ADAM10 levels rapidly to functional perturbations as well as to slower changes induced by aging and/or maturation.

### Transcriptional regulation of ADAM10

The human ADAM10 gene is localized on chromosome 15, whereas its murine homolog is found on chromosome 9 (Yamazaki et al., 1997a,b). Both genes are comprised of about 160 kb with high sequence preservation within the first 500 bp upstream of the translation initiation site (Prinzen et al., 2005). The human core promoter is positioned at −508 to −300 bp and contains no TATA box but several functional binding sites for common transcription factors such as Sp1 and USF (Prinzen et al., 2005). SNPs in the human promoter region at position −279 and −630 indicated no association with AD (Prinzen et al., 2005), whereas a SNP located at −644 was correlated with CSF APPs-alpha levels (Bekris et al., 2011). The 5′ UTR of the human gene was located 444 bp upstream of the start codon (Lammich et al., 2010), the 3′ UTR up to 1254 bp downstream of the stop codon (Augustin et al., 2012).

Even before the promoter of human ADAM10 was described, several pathways regulating the enzyme's expression had been identified: for example, in the prostate cancer cell line LNCaP insulin-like growth factor I combined with 5 alpha-dihydrotestosterone increased mature and immature ADAM10 protein amounts (McCulloch et al., 2004). Similarly, EGF led to the up-regulation of ADAM10 mRNA and protein in those cells. In addition, the Tcf/Lef-family of transcription factors which is known to interact with beta-catenin (Wisniewska, 2013) also seems to be involved: Wang et al. demonstrated in transgenic AD mice the induction of Wnt signaling by huperzine A. This was accompanied by elevated beta-catenin levels and increased ADAM10 protein levels (Wang et al., 2011). These findings were corroborated by the observation that NMDA receptor activation in primary neurons similarly increased ADAM10 via Wnt/MAPK signaling (Wan et al., 2012).

Using different cell systems Paired Box Genes (PAX) were similarly identified as putative ADAM10 regulators. In melanoma cells chromatin immunoprecipitation assay and overexpression as wells as siRNA-mediated knock-down gave evidence that PAX2 can regulate ADAM10 expression (Lee et al., 2011). Downregulation of PAX2 via siRNA in A498 (renal carcinoma), EAhy (endothelial), T98G (glioblastoma), and SKOV3ip (ovarian carcinoma) cells revealed a nearly total loss of ADAM10 protein as demonstrated by Western blot analysis (Doberstein et al., 2011). Therefore, PAX2 seems to play an important role in ADAM10 expression control—at least in cancer cells. Interestingly, the related PAX4 has been shown to regulate ADAM10 post-transcriptionally (see paragraph “Regulation of ADAM10 at the translational level”).

Another signaling pathway that increases ADAM10 amount within the cell via gene regulation requires melatonin. It has been reported that melatonin elevates ADAM10 level in HEK293 and neuronal SH-SY5Y cells via G protein-coupled receptor-induced PKC/Erk activation (Panmanee et al., 2015; Shukla et al., 2015). This effect seems to depend on human ADAM10 promoter region −1193 to −555 as a respective deletion construct failed to respond in a reporter gene assay (Shukla et al., 2015). The authors of the report discuss that the binding sites of cAMP response element-binding protein (CREB) and octamer-binding transcription factor 1 (Oct-1) which were described earlier (Prinzen et al., 2005) might contribute to the regulation or that a yet unidentified Hypoxia-inducible factor 1 (HIF-1) binding site might be responsible. The regulation of ADAM10 via the sleep hormone melatonin seems highly interesting as sleep disturbances are considered characteristic symptoms of AD (for example Sung et al., 2017).

Agonists specific for Peroxisome Proliferator-Activated Receptor alpha (PPARalpha) but not PPARbeta, delta, or gamma elevated ADAM10 protein amount in primary murine hippocampal neurons (Corbett et al., 2015). Seven PPAR responsible elements were identified by *in silico* analysis and the specific agonist GEM led to enrichment of PPARalpha and its heterodimer Retinoid X Receptor alpha (RXRalpha) binding partner at two direct repeat 1 PPAR responsive elements (PPRE) located in the ADAM10 promoter in wild type, but not in PPARalpha knock-out hippocampal neurons. 9-cis retinoic acid failed to synergistically increase ADAM10 amount in this context, therefore a non-permissive PPARalpha/RXRalpha heterodimer seems to regulate ADAM10 expression similar to the RARalpha/beta/RXR dimer from earlier investigations (Tippmann et al., 2009). PPARalpha is known to be involved in fatty acid metabolism. In this regard, it is of interest that lowering the cholesterol amount of cells increased ADAM10's catalytic but not transcriptional activity (Kojro et al., 2010) and that various fatty acids and lipids such as Docosahexaenoic acid (DHA) interfere with the balance of APP processing (e.g., Eckert et al., 2011; Grimm et al., 2016). Additionally, Sex-determining region Y-box 2 (Sox2), a major factor of adult tissue homeostasis and regeneration control, was recently identified to upregulate ADAM10 expression in HEK293 cells using overexpression experiments (Sarlak et al., 2016).

The retinoic acid receptor (RAR) family is particularly interesting with regard to ADAM10 regulation because of its therapeutic potential. Both, RAR alpha and beta are capable of inducing human ADAM10 promoter activity (Tippmann et al., 2009). Moreover, the commercially available drug acitretin which intracellularly liberates retinoic acid (Ortiz et al., 2013), shifts APP processing in AD model mice toward the alpha-secretase cleavage pathway (Tippmann et al., 2009). The neuroprotective property of RARalpha agonists has been shown in cortical cultures, an AD mouse model (Tg2576 mice) (Jarvis et al., 2010), as well as in hippocampal tissue of aged SAMP8 mice (Kitaoka et al., 2013). Cilostazol-stimulated N2A cells with overexpression of human mutated APP also displayed ADAM10 elevation which was significantly attenuated by a RARbeta inhibitor and RARbeta-gene silencing (Lee et al., 2014). The effect of cilostazol on ADAM10 expression could be antagonized by sirtinol and by Sirtuin 1 (SIRT1)-gene silencing, suggesting that RARbeta and this class of deacetlyase together act on the ADAM10 promoter.

**Figure 3:**
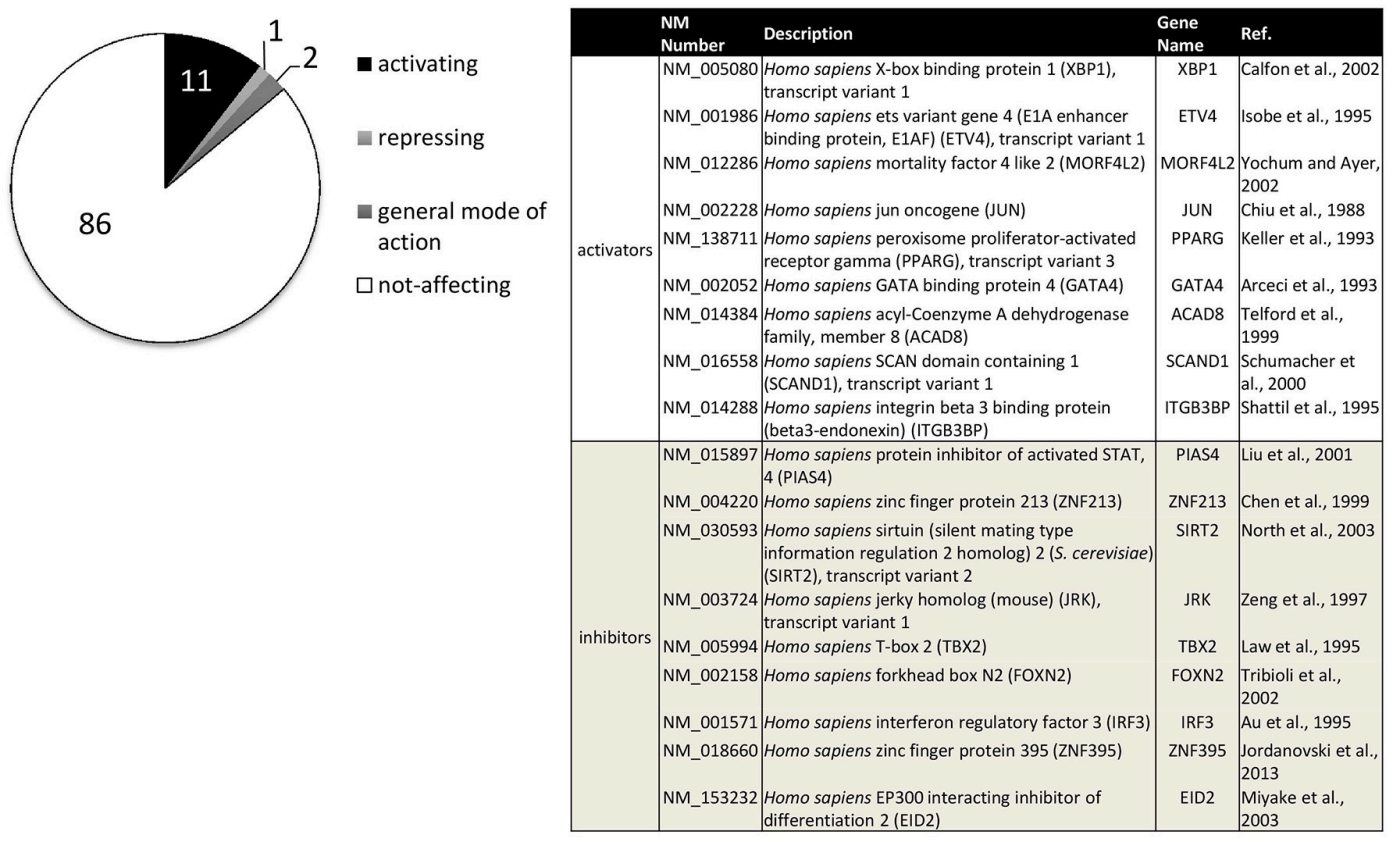
**Transcription factors influencing human ADAM10 promoter activity in SH-SY5Y cells**. Original data published in: Reinhardt et al. (2014). Factors filtered for effect size (promoter activity above 100+5xSD or below 100-2.5xSD of control) and reproducibility (SD≤ 15% of effect size in at least three independent experiments). Percentage of activating or repressing factors are indicated (left), factors with a general mode of action on transcription such as activator of basal transcription 1 (ABT1) were excluded. The table (right) shows the nine transcription factors with either strongest activating or inhibiting effects on the human ADAM10 promoter ranked from strongest to weakest.

For a systematic approach on transcription factors relevant to ADAM10 regulation, we performed a screening approach (Reinhardt et al., 2014). Figure 3 sums up transcription factors that showed a significant influence on ADAM10 expression in these investigations. One has to consider that the screening approach was performed in human neuronal SH-SY5Y cells and only included single expression plasmids for 704 human transcription factors. Therefore, accessory proteins for single factors might not have been present in the cell line or combinations of transcription factors might be needed for full activation. However, we identified 11% transcription factors with a comparably strong influence on promoter activity of ADAM10 with nine factors inhibiting and 74 factors increasing transcriptional activity (Figure 3). Starting from this screening we were able to further characterize regulation of ADAM10 via one of the strongest inducers—X-Box binding protein 1 (XBP-1, Calfon et al., 2002). The active transcription factor is built upon ER stress sensor Inositol requiring enzyme 1 alpha (IRE1 alpha) activation and leads to increase in ADAM10 mRNA as well as protein and subsequent release of the APP cleavage product APPs-alpha (Reinhardt et al., 2014). Interestingly, we also found the amount of XBP1-mRNA to be decreased in Alzheimer model mice at higher age and also in Alzheimer's disease patients.

### Epigenetic regulation of ADAM10

Currently, little is known about the epigenetic regulation of ADAM10. The 5′-untranslated region of the human ADAM10 gene contains a large GC-rich domain at −700 to +200 bp. The GC content of the first 600 bp upstream of the ATG of the human ADAM10 gene is 67% and nine CpG islands have been predicted (Prinzen et al., 2005). This abundance of CpGs suggests that cytosine methylation could play a role in regulating the proteinases' expression. SIRT1 an evolutionarily conserved NAD^+^-dependent deacetylase pivotal for metabolic control has been identified to increase ADAM10 expression (Lee et al., 2014). SIRT1 is involved in histone deacetylation and methylation, promoter CpG island methylation, and inactivation of non-histone transcription factors (Zhang and Kraus, 2010). Conceivably, SIRT1 is also involved in deacetylation of RAR or in chromatin modifications upon recruitment by the receptor but currently this has not yet been demonstrated. Investigations into these regulatory mechanisms are non-trivial and complicated by the fact that SIRT1 also acts on the cellular retinoid binding protein II (CRABPII) and also has a more general effect on RA signaling (Tang et al., 2014).

In transgenic AD model mice (5 × FAD) a significant increase in global DNA methylation, measured by 5-methyl cytosine, has been reported and additional changes in e.g., demethylase Dnmt3b or enzymes of histon acetylation/ deacetylation such as Hdac2, Jarid1a, or G9a (Griñán-Ferré et al., 2016). Surprisingly, no changes of ADAM10 expression were observed when using whole brain mRNA preparations. Although ADAM10 was found within the top CpG sites of an epigenomic analysis of psychiatric tic-diseases using peripheral blood samples (cg00785856, Zilhão et al., 2015), the methylation site did not reach significance at the genome-wide threshold.

Finally, as melatonin seems to be able to increase the level of deacetylase in young and aged primary neurons (Tajes et al., 2009), the observed induction of ADAM10 by melatonin (Panmanee et al., 2015; Shukla et al., 2015) might also rely on deacetylase activation.

### Regulation of ADAM10 at the translational level

Besides regulation on the transcriptional/epigenetic level, translational modifiers can regulate the amount and availability of ADAM10: RNA structure, RNA-binding proteins (RBPs), and miRNAs have been reported to play a role.

The working group of Christian Haass explored a suppression of ADAM10 expression by its 5′UTR (Lammich et al., 2010) and identified a stable G-quadruplex structure of ADAM10 mRNA (Lammich et al., 2011). The stability of a G-quadruplex structure depends in part on binding proteins, such as fragile X mental retardation protein (FMRP; Oostra and Willems, 1995) and indeed, FMRP immunoprecipitated from cortical mouse tissue revealed bound ADAM10 mRNA (Pasciuto et al., 2015). Mice lacking FMRP displayed a shift of APP processing toward the non-amyloidogenic pathway during early stages of development, which subsequently led to synaptic and behavioral deficits (Pasciuto et al., 2015). Lack of FMRP could increase ADAM10 levels because FMRP stabilizes the G-quadruplex structure and can thus perturb translation initiation, as has been previously suggested for two other mRNAs (MAP1B and PP2A) that are FMRP targets (Lu et al., 2004; Castets et al., 2005). Another RNA-binding protein found to regulate ADAM10 is the neuronal ELAV protein: nELAV was shown by using immunoprecipitation to bind ADAM10 mRNA via an adenine- and uridine-rich element (Amadio et al., 2009). This might result in an increase in amyloidogenic APP processing. Since A-beta peptides have been found to inhibit ELAV-binding to ADAM10 mRNA (Amadio et al., 2009), this could reduce the ADAM10 amount even further, potentially leading to a vicious cycle.

miRNAs can silence cytoplasmic mRNAs either by triggering degradation or by promoting translation repression. For ADAM10 a prominent example for such a regulatory mechanism is hepatic miR-122, which decreased ADAM10 protein in human hepatic cancer cell lines (Bai et al., 2009). Using a systematic approach, i.e., a combination of different bioinformatics tools, we identified several candidate miRNAs that should act on ADAM10 and evaluated three of them via reporter gene assay—miR-103, -107, and -1306 (Augustin et al., 2012). Additionally, miR-144/451 which has been shown to be induced by A-beta peptide in SH-SY5Y cells decreased ADAM10 protein amount (Cheng et al., 2013). This regulation might be indirect and based on the transcription factor PAX4 (Zhang et al., 2015). In gastric cancer tissue miR-448 (Wu et al., 2016) and in tumor initiator cells of head and neck squamous cell carcinoma miR-494 (Chang et al., 2015) were also identified as novel regulators of ADAM10.

### Post-translational regulation of ADAM10: maturation and interaction partners

After their synthesis, membrane proteins mature along the secretory pathway; they are transported to distinct compartments of the cell and finally, they locally interact with proteins and lipids of the phospholipid-bilayer. Eventually, they are degraded. Protein synthesis and removal are in homeostasis and thus determine the concentration of functional intramembranous proteins. In principle, ADAM10 can be regulated at all of these stages, offering possibilities for intervention.

The ADAM10 zymogen is cleaved by proprotein convertases within the secretory pathway to yield the active enzyme (see paragraph 1). Removal of the prodomain of ADAMs likely involves a canonical consensus site for the proprotein convertase Furin (Roebroek et al., 1994), which is located between the pro- and the catalytic domain of ADAM10 (Anders et al., 2001). More recently, a novel cleavage site upstream of the prodomain has been identified (Wong et al., 2015). ADAM10 has four potential N-glycosylation sites of which three are located in the metalloprotease domain (N267, N278, and N439) and one in the disintegrin domain (N551). In bovine ADAM10 all four have been found glycosylated and required for full *in vivo* activity (Escrevente et al., 2008).

Binding of ADAM10 to synapse associated protein 97 (SAP97) is required for inserting ADAM10 into the synaptic membrane (Marcello et al., 2013). Interaction of SAP97 with ADAM10 is mediated via a protein kinase C (PK C) phosphorylation site within the SAP97 SRC homology domain (Saraceno et al., 2014). Removal of ADAM10 from excitatory synapses occurs by clathrin-mediated endocytosis in human hippocampal tissue (Marcello et al., 2013). This is mediated by the clathrin adaptor protein AP2 which interacts with the ADAM10 C-terminal domain. In addition to control of surface concentrations of ADAM10 by transport mechanisms, further cleavage events may occur: the ectodomain of ADAM10 can be processed by ADAM9/15 or gamma-secretase (Cissé et al., 2005; Parkin and Harris, 2009; Tousseyn et al., 2009). Using recombinant mouse ADAM9 prodomain as a competitive inhibitor of ADAM9, Moss et al. demonstrated an increase of ADAM10-dependent APP processing in human neuronal SH-SY5Y cells (Moss et al., 2011). However, a truncated soluble ADAM10 construct was incapable of shedding cell-associated amyloid precursor protein while earlier reports described that shedded ADAM10 had the ability to cleave endogenous Prion protein in fibroblasts (Cissé et al., 2005).

The intensity of ADAM10 cleavage may further depend on the cytoskeleton: a dominant negative dynamin I mutant not only increased surface expression of both, immature, and mature ADAM10 but also strongly increased the amount of the C-terminal cleavage product of ADAM10 (Carey et al., 2011). In addition to its role as a protease acting at the cell surface it has been speculated that the soluble ADAM10 C-terminus could act as a signaling molecule, facilitating nuclear entry of other proteins (Endsley et al., 2014). A protein class which is deeply involved in for example cytoskeletal anchoring and protein-trafficking is the tetraspanin family (Charrin et al., 2014). Several tetraspanins have been identified to interact with ADAM10: tetraspanin 12 (Tspan 12) binds to ADAM10 in a palmitylation-dependent mechanism and increases non-amyloidogenic shedding of APP by increased enzymatic maturation of the protease (Xu et al., 2009). Co-immunoprecipitation experiments also identified specific ADAM10 interactions with Tspan5, Tspan10, Tspan14, Tspan15, Tspan17, and Tspan33/Penumbra (Haining et al., 2012), which all led to enhanced enzyme maturation. Interestingly, only overexpression of Tspan15 resulted in a reduction of ligand-induced Notch-1 processing by ADAM10 (Jouannet et al., 2016). This led to the assumption that the tetraspanins might differentially influence compartimentalization of ADAM10. Indeed, the apparent diffusion coefficient of ADAM10 was higher in cells overexpressing Tspan15 as compared to control cells or Tspan5 overexpressing cells and also decreased the co-immunoprecipitation of proteins of the tetraspanin web with ADAM10 (Jouannet et al., 2016). Tspan12 and 17 also seem to stabilize a high molecular weight protein complex that tethers ADAM10 to the gamma-secretase allowing rapid sequential processing of substrates (Chen et al., 2015).

ADAM10 is known to be mainly located outside of lipid rafts and alpha-secretase cleavage of APP occurs in non-raft domains (Kojro et al., 2001). Targeting ADAM10 artificially into lipid raft domains of the plasma membrane resulted in impaired enzymatic activity in human neuroblastoma cells (Harris et al., 2009; Kojro et al., 2010). Depletion of one of the constituents of lipid rafts, i.e., cholesterol, enhanced ADAM10 activity in different cellular models (Kojro et al., 2001, 2010; Matthews et al., 2003). The sigma-1 receptor contains a cholesterol recognition domain in its C-terminus and is able to remodel lipid rafts by changing the relative distribution of cholesterol between raft and non-raft fractions (Takebayashi et al., 2004). Interestingly, overexpression of sigma-1 in HEK293 or COS cells diminished Betacellulin cleavage by ADAM10 further substantiating the lipid-sensitivity of the enzyme (Li et al., 2012). Several investigations also report on influence of different lipid species such as trans fatty acids on APP processing balance (e.g., Eckert et al., 2011; Grimm et al., 2012) but in this regard it is not clear if this has a direct influence on ADAM10 or whether indirect mechanisms are involved.

## Roles of ADAM10 in neural homeostasis and pathology

ADAM10 has a number of physiological functions (see above) contributing to brain development or neural homeostasis. Diseases challenge this physiological state and the brain reacts to such perturbations with adaptations at the molecular, cellular, and functional level. The picture that is currently emerging from studies using animal models and human brains suggests a two-faced role of ADAM10 in diseases: beneficial as well as detrimental effects can be attributed to the protease depending on the specific setting and the substrates involved. In the following we review some of the conditions and diseases in which ADAM10 has been implicated.

### ADAM10 in aging and alzheimer's disease

ADAM10's role in these contexts is of particular interest because of its function as *in vivo* alpha secretase (Jorissen et al., 2010; Kuhn et al., 2010). Cleavage of APP along the non-amyloidogenic pathway yields APPs-alpha, which is important for neuroprotection (Kögel et al., 2012), learning and memory (Taylor et al., 2008; Hick et al., 2015; Xiong et al., 2016), and the structural integrity of neurons (Lee et al., 2010; Tyan et al., 2012; Weyer et al., 2014; Hick et al., 2015). Because cleavage of APP along the non-amyloidogenic pathway decreases with aging (Kern et al., 2006) and reduced APPs-alpha levels were found in CSF of some AD patients (Lannfelt et al., 1995; Sennvik et al., 2000), it is likely that insufficient APPs-alpha levels could contribute to the cognitive deficits of AD patients.

What is known about age-dependent changes in ADAM10 levels or activity in human brain? Unfortunately, with the notable exception of a publication from Bernstein et al. (2003) who compared still-born children with normal aged adults and found an increase in ADAM10 amount, data on ADAM10 in human brain are scarce. To study ADAM10 in humans, peripheral surrogate markers have been used, although it is unclear how comparable they are to CNS expression levels. A study aiming at comparing brain and leukocyte APP processing reported that while ADAM10 is present in brain it remains undetectable in the blood leukocyte fraction (Delvaux et al., 2013). Others, however, demonstrated ADAM10 expression in peripheral mononuclear blood cells as well as in platelets (Colciaghi et al., 2002). Using three groups of cognitively healthy subjects, we recently described an elevation of ADAM10 protein amount as well as catalytic function with cognitively healthy aging (Schuck et al., 2016). The reason why ADAM10 should be up-regulated is unclear. It is conceivable that it is a reaction to age-dependent changes in stress signatures (such as ER stress; e.g., Taylor, 2016) and thus represents a protective response. Although more data are needed, “healthy agers” show an ADAM10-increase whereas AD patients show a decrease (see below). In the former case APPs-alpha could be present in sufficient amounts protecting the brain whereas in the latter case APPs-alpha levels might be insufficient.

Using animal models of AD the role of ADAM10 as a protective protease has been demonstrated: overexpression of the protease at low level (30% above endogenous expression) was sufficient to nearly abolish plaque deposition in APP/PS1 AD model mice (Postina et al., 2004). These changes went hand-in-hand with improvements of learning and memory. In line with this gain-of-function approach, overexpression of a dominant negative ADAM10 mutant reduced alpha-secretase activity and worsened cognitive deficits (Schmitt et al., 2006). Interestingly, investigations using peripheral platelets of AD patients and healthy controls reported a decreased ADAM10 amount in AD patients (Colciaghi et al., 2002). Furthermore, ADAM10 levels in patient platelets were highly correlated with performance of the patients in psychological tests (Manzine et al., 2013, 2014). Together, these data suggest that normalizing or even increasing ADAM10 levels in AD could have a disease-modifying or at least disease-protracting effect.

Finally, it should be kept in mind that ADAM10 is multifunctional and that some effects of ADAM10 in the context of aging and AD could depend on ADAM10-mediated cleavage of other substrates than APP, such as Klotho (Chen et al., 2007; Bloch et al., 2009). This protein is linked to longevity (Kurosu et al., 2005) and soluble Klotho (s-Klotho) may be cardioprotective (Xie et al., 2012). Lower CSF s-Klotho levels have also been associated with endothelial dysfunction and neuronal damage in neuropsychiatric systemic lupus erythematosus patients (Ushigusa et al., 2016). Thus, lower ADAM10 levels in the aged brain may have detrimental effects on several levels involving APP processing as well as the processing of other ADAM10 substrates.

### ADAM10, dendritic spines and fragile X syndrome

The level and/or activity of ADAM10 affect neuronal structures in the adult brain, in particular dendritic spines. This was shown using conditional ADAM10-deficient mice (Prox et al., 2013), which exhibited hippocampal neurons with fewer and abnormally shaped spines. The effect of ADAM10 on spines may depend on several substrates involved in the regulation of spine density, geometry and dynamics, including APP, N-cadherins, Neurexins, Neuroligins, and Nectin-1 (Prox et al., 2013). These substrates act as cell adhesion molecules and are known to influence spine morphology as well as synaptic transmission.

Of particular interest in this context is again the link between ADAM10 and APP. APP and in particular its cleavage product APPs-alpha have been shown to regulate dendritic complexity as well as spine numbers of hippocampal neurons (Lee et al., 2010; Tyan et al., 2012; Weyer et al., 2014). This effect appears to be age-dependent: whereas young APP-deficient mice had normal spine numbers, older APP-deficient mice showed a decrease in their spine density (Tyan et al., 2012). It may also depend on the brain region, since APP levels may show regional variations (Del Turco et al., 2016). Since APPs-alpha is generated by ADAM10 cleavage of APP, it is likely that some of the structural effects on spines seen in conditional ADAM10 knock-out mice (Prox et al., 2013) are the result of reduced APPs-alpha levels. Indeed, Prox et al. (2013) reported a reduction of APPs-alpha in brain of conditional ADAM10 knock-out mice to 5% of control levels. Since aging is also associated with reduced dendritic complexity and spine densities (Dickstein et al., 2007), it is attractive to speculate that reduced ADAM10 levels/activity and reduced APPs-alpha levels could play a role in this context (Lannfelt et al., 1995; Sennvik et al., 2000).

Whereas, reduction of ADAM10 may contribute to conditions in which fewer dendritic spines are observed, too much ADAM10 could contribute to diseases with the opposite phenotype, i.e., too many spines. Fragile X syndrome (FXS) is a good example for such a disease and is characterized by increased spine numbers and abnormally long spines. Mice with a fragile X mental retardation protein (FMRP) knock-out at an early adult age (P21, Pasciuto et al., 2015) showed a parallel increase in the expression of APP and mature ADAM10, suggesting that ADAM10 processing of APP could play a role. Indeed, primary fibroblasts obtained from adolescent and adult patients with FXS showed similar changes (Pasciuto et al., 2015), suggesting that an upregulation of ADAM10 and APP could also occur in brains of FXS-patients. In line with these findings, overexpression of APP (Lee et al., 2010) caused FXS-like spine changes *in vitro*.

In sum, under healthy conditions ADAM10 and its processing of cell adhesion molecules at synapses is in a homeostatic balance. Reduction of ADAM10 levels may cause a reduction in spine densities. Conversely, an increase in ADAM10 levels may increase the density of spines. Normalizing ADAM10 levels could be a potential therapeutic strategy.

### Synaptic function and epilepsy

Dendritic spines and excitatory synaptic neurotransmission are intimately linked (Kasai et al., 2010). It is, therefore, in line with the effects of ADAM10 on dendritic spines that conditional ADAM10 knock-out mice show functional abnormalities at excitatory synapses: electrophysiological analysis of hippocampal CA1 neurons revealed almost normal basal synaptic transmission and short-term-plasticity but a grossly impaired induction of long-term-potentiation (Prox et al., 2013). These electrophysiological abnormalities were accompanied by reduction of postsynaptic density protein-95 (PSD-95) and several NMDA-receptor subunits, suggesting a severe disruption of synaptic architecture and function. Spatial learning was impaired at the behavioral level (Prox et al., 2013). Mechanistically, the impairment of synaptic plasticity and learning could be linked to several of the substrates of ADAM10 at the synapse. Again, APP is one of the more interesting candidates because its fragment APPs-alpha has been shown to be involved in synaptic plasticity, as well as learning and memory in the hippocampus (Taylor et al., 2008; Hick et al., 2015). At present it is unknown whether some of the abnormalities of the conditional ADAM10 mice could be rescued by recombinant APPs-alpha. Answering this question could help to better understand the relative importance of APP in this context.

Gain-of-function experiments resulted in an increased susceptibility of neurons for seizures: in mice overexpressing ADAM10 under the Thy1 promoter (~postnatal day 1), kainate-treatment evoked stronger and longer episodes of seizures as compared to wild type mice (Clement et al., 2008). Moreover, a dominant negative variant of ADAM10 seemed protective against this form of experimental epilepsy as shown e.g., by decreased neuronal damage score. The role of ADAM10 in epilepsy is complex, however, since conditional ADAM10 knock-out mice also showed seizures. However, these seizures may be linked to the gliosis observed in these mice (Prox et al., 2013). Thus, different disease mechanisms could play a role and additional work is needed before the role of ADAM10 in epilepsy can be assessed.

### ADAM10 and traumatic brain injury

**Figure 4:**
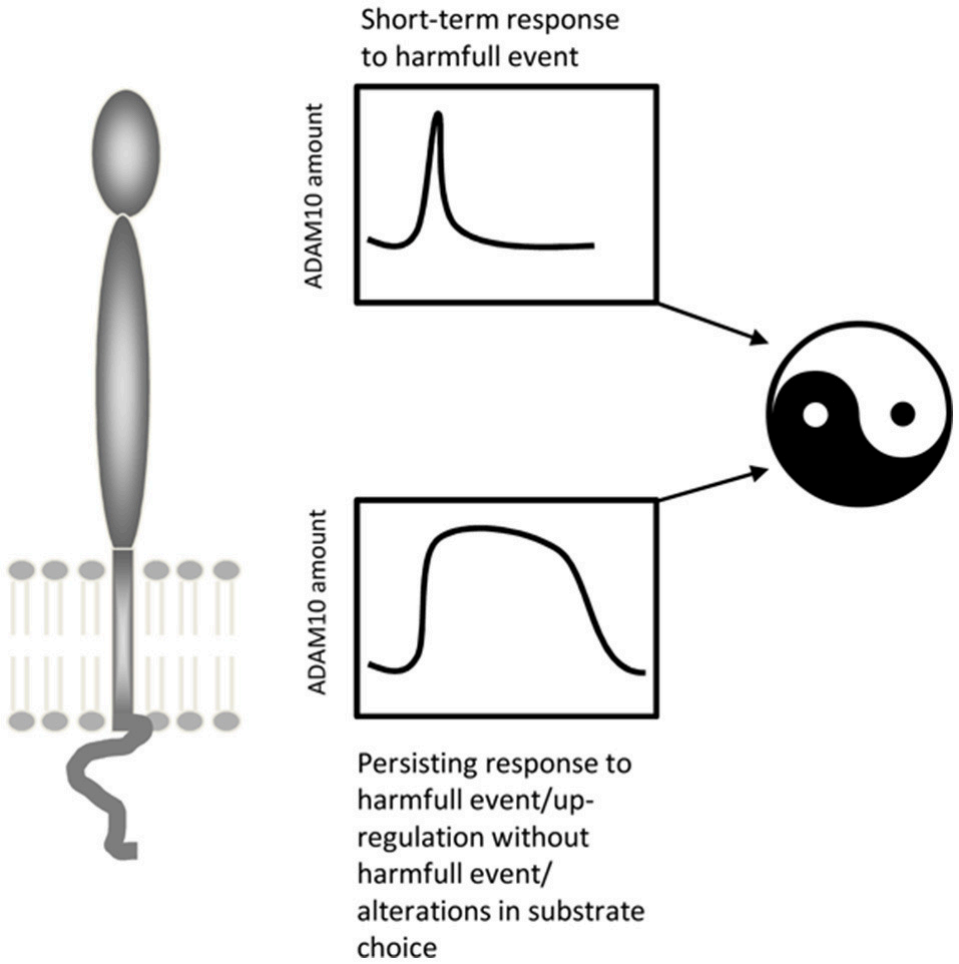
**ADAM10's potential two-faced role under conditions of brain injury**. Whereas a transiently increased activity/amount of ADAM10 seems to be part of a protective and restorative response to mild neural lesions, a persistent upregulation of ADAM10 as seen following severe lesions may be deleterious.

ADAM10 is upregulated at injury sites (Zohar et al., 2011) and in denervated areas of the brain following brain injury (Warren et al., 2012; Del Turco et al., 2014). Reactive astroglia but not microglia has been shown to upregulate the protease following denervation (Warren et al., 2012; Del Turco et al., 2014). ADAM10's role in traumatic brain injury is still poorly understood and different modes of action have been proposed, which may not be mutually exclusive. First, the protease could be involved in the reorganization of the extracellular matrix of denervated regions (e.g., Deller et al., 2000), which may be a requirement for denervation-induced synaptic reorganization to occur (Warren et al., 2012). Secondly, ADAM10 could process synaptic adhesion molecules such as N-cadherin (Malinverno et al., 2010; Warren et al., 2012), neuroligins (Suzuki et al., 2012), or ephrins (Janes et al., 2005), which could tether degenerating terminals to their postsynaptic membranes. Cleaving the transsynaptic molecular bridge could be a necessary first step for re-innervation. Thirdly, ADAM10 could cleave APP and liberate APPs-alpha (Del Turco et al., 2014), which is neuroprotective *in vitro* (Kögel et al., 2012) and protects neurons *in vivo* following brain injury (reviewed in: Plummer et al., 2016). Of note, in these contexts upregulation of ADAM10 is associated with a short-term neural “defense”-reaction. This reaction seems to be transient and ADAM10 levels return to normal within a few days. In contrast, under lesioning conditions resulting in long-term upregulation of ADAM10 (Warren et al., 2012) synaptic reorganization failed and functional deficits persisted. Under these conditions, pharmacological blockade of ADAM10 helped to restore function, suggesting that long-term upregulation of ADAM10 is detrimental for brain rewiring. Collectively, these findings suggest the following model for ADAM10's role in brain injury: ADAM10 plays a plasticity-enhancing and neuroprotective role during the first phase following injury. It shapes the extracellular environment for sprouting fibers, clears synaptic sites, and liberates neuroprotective APP fragments. During the second phase, however, sprouting of surviving fibers occurs and new synapses form. If ADAM10 is still upregulated at this time point it could interfere with the stabilization of new synapses by cleaving the molecular bridge that binds pre- and postsynaptic structures (Figure 4).

### Stroke and psychiatric diseases

A positive association between the rs653765 polymorphism of ADAM10 and atherosclerotic cerebral infarction has been found in a Chinese population cohort (Li et al., 2013). Patients that carried the rs653765 C > T mutation also showed increased ADAM10 mRNA in PBMCs as did aged patients in comparison to younger patients or healthy controls (>70 years). As already mentioned, an association between CpG-site methylation in the ADAM10 locus and psychiatric tic-disorders has been identified (Zilhão et al., 2015). Beside this epigenetic association, ADAM10 has been characterized as one of the candidates within a low density GWA study for conduct disorder (Jian et al., 2011). Further associations with psychiatric disorders are conceivable, since ADAM10 processes neuroligins, which have also been identified as candidate genes in autism spectrum disorders and schizophrenia (e.g., Sun et al., 2011; Chen et al., 2014). In this regard it is of interest that Ray and colleagues recently reported alterations of APP processing and amount not only in FXS patients but also in autism spectrum disorder patients (Ray et al., 2016). However, they also reported age-dependent elevation of ADAM17 in the latter so that this protease might be due to observed changes instead of ADAM10.

### Brain tumors

ADAM10 may have deleterious effects for patients with brain tumors because it may promote the spreading of tumor cells. Reduced motility of glioblastoma cells treated with ADAM10-targeted siRNA has been observed (Kohutek et al., 2009) and invasiveness of pituitary adenomas correlated with ADAM10 expression level (Pan et al., 2012). Both publications suggests that ADAM10 may process putative barriers restricting tumor cells. With regard to cancer stem cells Bulstrode et al. reported that ADAM10 promotes the self-renewal of brain tumor sphere forming cells (Bulstrode et al., 2012). Additionally, treatment with inhibitors specific for ADAM10 or ADAM17 increased immune recognition of glioblastoma-initiating cells by natural killer cells (Wolpert et al., 2014). This seemed to be due to enhanced cell surface expression of UL16-binding protein 2 (ULBP2), which is shedded by both proteinases. In sum, stimulating ADAM10 expression, as suggested for AD patients (see below), may not be an option for oncology patients.

## Conclusion and outlook—ADAM10-targeting drugs as novel therapeutics?

ADAM10 is a biologically multifunctional protease involved in many important processes. It is expressed almost ubiquitously in the body. High amounts of ADAM10 are found in neural tissue during development, maturation and aging. Under conditions of neuronal activity and under some pathological conditions, ADAM10 expression is altered which, in turn, leads to changes in the processing of its substrates. The biological activity of these substrates and their cleavage products lead to measurable changes in function, biochemistry and even neural structures.

How can ADAM10 be considered a target for therapy in spite of the large number of substrates with multiple functions? First of all it has to be kept in mind that the majority of data on ADAM10 was obtained using *in vitro* systems. Although these studies can show putative interactions, such *in vitro* interactions require *in vivo* verification. ADAM10 can only cleave putative substrates if protease and substrate are in the same microcompartment at the same time. Since the availability of substrates and their distribution changes during development and aging, it is likely that changes in ADAM10 expression result in different effects depending on the age and stage of development of an organism. Of particular importance for the use of drugs targeting ADAM10 is the fact that ADAM10 shows an increasing overlap with its substrate APP with age (Marcinkiewicz and Seidah, 2000), suggesting that ADAM10-mediated APP cleavage may become more relevant at later stages in life. Regardless of these considerations, a rational approach to therapy development will take all these possibilities into account and will look at the net biological effects changes in ADAM10 expression induce in neural tissue. Complex *in vitro* systems, such as organotypic slice cultures (e.g., Gähwiler et al., 1997; Del Turco and Deller, 2007) and *in vivo* models (e.g., Postina et al., 2004) will help to address these questions.

The duration of ADAM10 expression changes may also play a critical role during the course of a disease. The enzyme can be briefly upregulated or persistently increased, depending on the specific conditions. Thus, ambivalent or even opposite outcomes can be expected for ADAM10 effects on brain structure and function, as has been shown for its role in brain injury (see Figure 4). Finally, patients may have different genetic predispositions or constitutively elevated ADAM10 levels, which might also harm the brain as has been shown for infarction and cancerogenesis (Pan et al., 2012; Li et al., 2013).

In sum, there are drug safety-issues which need to be explored before ADAM10 targeting drugs can be considered for therapy. The complex expression patterns and time courses of ADAM10 and its substrates may constrain the use of ADAM10-targeting drugs to specific situations, aged patients or some diseases. A clinical pilot study using acitretin was, however, promising (Endres et al., 2014). Acitretin (Neotigason), which increases APP processing along the non-amyloidogenic pathway *in vitro*, in primary cells, and in AD model mice (Tippmann et al., 2009; Reinhardt et al., 2016), was given to patients with mild to moderate AD for 4 weeks. Compared to the placebo group, treated patients showed a significant increase in their CSF APPs-alpha levels. Acitretin-treatment was well-tolerated and considered overall safe (Endres et al., 2014). Longer and larger trials will now be needed to evaluate the potential of acitretin as a novel AD-therapeutic. In any case, the pilot study raises hopes that at least for some AD patient groups ADAM10-targeting therapies may eventually prove to be useful.

## Author contributions

KE and TD contributed equally to all aspects of this review, including development of the overall concept, writing and creating the figures.

## Funding

Grant sponsor: Deutsche Forschungsgemeinschaft (DFG, FOR 1332 to TD) and (NGFN, FKZ01GS08130) and the Alfons Geib-Stiftung to KE.

### Conflict of interest statement

The authors declare that the research was conducted in the absence of any commercial or financial relationships that could be construed as a potential conflict of interest.
